# Stimulus Onset Modulates Auditory and Visual Dominance

**DOI:** 10.3390/vision4010014

**Published:** 2020-02-29

**Authors:** Margeaux F. Ciraolo, Samantha M. O’Hanlon, Christopher W. Robinson, Scott Sinnett

**Affiliations:** 1 College of Health Solutions, Arizona State University, 550 N 3rd St., Phoenix, AZ 85004, USA; 2 School of Psychological Science, Oregon State University, 2950 SW Jefferson Way, Corvallis, OR 97331, USA; 3 Department of Psychology, The Ohio State University at Newark, 1179 University Dr., Newark, OH 43055, USA; robinson.777@osu.edu; 4 Department of Psychology, University of Hawai’i at Mānoa, 2530 Dole St., Sakamaki C400, Honolulu, HI 96822, USA; ssinnett@hawaii.edu

**Keywords:** multisensory integration, sensory dominance, multisensory processing, cognition, auditory perception, visual perception

## Abstract

Investigations of multisensory integration have demonstrated that, under certain conditions, one modality is more likely to dominate the other. While the direction of this relationship typically favors the visual modality, the effect can be reversed to show auditory dominance under some conditions. The experiments presented here use an oddball detection paradigm with variable stimulus timings to test the hypothesis that a stimulus that is presented earlier will be processed first and therefore contribute to sensory dominance. Additionally, we compared two measures of sensory dominance (slowdown scores and error rate) to determine whether the type of measure used can affect which modality appears to dominate. When stimuli were presented asynchronously, analysis of slowdown scores and error rates yielded the same result; for both the 1- and 3-button versions of the task, participants were more likely to show auditory dominance when the auditory stimulus preceded the visual stimulus, whereas evidence for visual dominance was observed as the auditory stimulus was delayed. In contrast, for the simultaneous condition, slowdown scores indicated auditory dominance, whereas error rates indicated visual dominance. Overall, these results provide empirical support for the hypothesis that the modality that engages processing first is more likely to show dominance, and suggest that more explicit measures of sensory dominance may favor the visual modality.

## 1. Introduction

Understanding how the mind creates stable and complete percepts out of seemingly distinct sensory experiences is an important scientific undertaking. Research indicates that various factors influence whether sensory events are perceived to belong to the same unitary percept [1,2]. In some situations, information arriving at separate senses can compete for resources, at times leading one event to be processed/perceived at the expense of another [3,4], a phenomenon predominantly referred to as *sensory dominance*.

The vast majority of research on sensory dominance has focused on audition and vision (although, see [5,6] for visuotactile and audiotactile examples, respectively). Overall, studies demonstrate that visual information tends to disrupt the processing of auditory information [7]. In seminal research by Colavita [3], participants were given a speeded task in which unimodal auditory, unimodal visual, or bimodal audiovisual targets were presented. Participants responded to each of the unimodal target types with different keys, and with both keys for bimodal targets. Interestingly, when presented with a bimodal target, participants almost exclusively responded to the visual component only (i.e., 98% of responses to bimodal targets were “visual only” in Experiment 1). Accordingly, the failure to respond to the non-visual modality of bimodal stimuli has often been referred to as the *Colavita visual dominance effect*. The Colavita effect occurs across a wide range of stimulus manipulations, including variations in stimulus intensity [3], modality [5,6], complexity [7,8], and spatial origin [3,9]; for a detailed review of the Colavita effect, see [10].

Examples of successful modulations of the Colavita effect are rare. Two such examples suggest that the Colavita effect may be susceptible to the *law of prior entry* [11,12] (see also [13] for discussion of a similar mechanism), which posits that sensory experiences that are being attended to are perceived more rapidly. In a variation of the task used by Colavita [3], Sinnett et al. [8] biased attention towards the auditory modality by increasing the frequency of unimodal auditory targets, but only observed a reduction in the Colavita effect (i.e., fewer “visual only” responses to bimodal targets, but no evidence of auditory dominance). Using a temporal order judgement (TOJ) task, Koppen and Spence [14] found that the Colavita effect was reversed only when the stimulus onset asynchrony (SOA) was outside the asymmetrical temporal window of audiovisual integration (i.e., the auditory stimulus was presented at least 65 ms ahead of the visual stimulus). Therefore, for this bias toward the visual modality to be eliminated, the primacy of the auditory stimulus must be obvious. 

Given the breadth of research demonstrating visual dominance, and the findings that biasing attention towards the auditory modality reduces visual dominance but does not reverse it to auditory dominance, the Colavita visual dominance effect appears fairly robust. Therefore, it may seem that auditory dominance simply does not occur in adults unless precise and extreme experimental conditions are met (but see [15,16,17,18,19] for sensory dominance shifts from auditory to visual dominance during development). However, there are limited situations in which audition has been shown to dominate vision [20,21,22,23]. In a study conducted by Robinson and colleagues [21], participants monitored a stream of bimodal audiovisual stimuli and were instructed to press a key in response to any deviations from a standard (i.e., respond to a unimodal auditory oddball (only the tone changed), a unimodal visual oddball (only the picture changed), or a bimodal oddball (both the tone and the picture changed)). Participants completed this basic *oddball detection task* under three response conditions: in the 1-button task, participants were instructed to press a single response key if they detected an oddball of any type (Experiment 1); in the 3-button task, participants were instructed to press separate keys for each type of oddball (Experiment 2); and in the modified 1-button task, participants were instructed to press the same key in response to unimodal oddballs and to refrain from responding to bimodal oddballs (Experiment 3). Interestingly, clear evidence of auditory dominance was demonstrated in both versions of the 1-button task, while visual dominance was observed in the 3-button task. To explain this pattern of results, Robinson et al. [21] theorize that auditory and visual dominance may be driven by two separate underlying mechanisms that engage at different times when observing and responding to bimodal audiovisual stimuli. Specifically, they posit that the auditory mechanism occurs earlier in processing and can disrupt visual stimulus encoding. By comparison, visual dominance arises later in the course of processing, when the participant is deciding on and executing a response.

It is important to note that the reversal of dominance types demonstrated by Robinson et al. [21] was the first reported example of auditory dominance in adults using this paradigm (see [20] for an example of auditory dominance in a modified n-back task). Given that it is not entirely clear what is driving some of the aforementioned findings, further examination of the stimulus factors influencing sensory dominance is critical to understanding this phenomenon. The oddball paradigm employed by Robinson et al. [21] is an ideal starting point for the examination of these factors because both auditory and visual dominance can be demonstrated with relatively small manipulations to experimental protocols (i.e., by simply changing the response options; with auditory dominance being demonstrated in the 1-button version of the task and visual dominance in the 3-button version).

Our research further examines sensory dominance within the context of the oddball paradigm utilized by Robinson et al. [21] by systematically manipulating stimulus onset asynchrony (SOA) in 1-button (Experiment 1) and 3-button (Experiment 2) versions of this task. As previously discussed, Robinson et al. [21] argue that the mechanisms driving sensory dominance may differ in their relative timing, with the auditory mechanism occurring earlier in processing (resulting in the disruption of visual stimulus encoding) and the visual mechanism occurring later in processing when the participant is making a response. Support for this argument has come from different experimental contexts. For example, auditory stimuli can slow down first fixations to visual stimuli [24,25] and also delay the visual P300 [26], suggesting that the auditory modality disrupts visual encoding. Furthermore, visual dominance effects occur more frequently when the participant must distinguish between the auditory and visual modality (e.g., “press button 1 for an auditory stimulus and button 2 for a visual stimulus” [3,10]). Additionally, Posner et al. [27] suggest that visual dominance may be a result of a generalized visual response bias that is intended to compensate for the reduced ability of visual changes to capture attention, compared to auditory changes. Taken together, these findings led us to hypothesize that, in the 1-button task, we would observe auditory dominance when the auditory stimulus was presented first, because it would disrupt visual encoding, and when the stimuli were presented simultaneously, because participants had only one response option. In regard to the 3-button task, we hypothesized that the need to decide between three possible response options would override the influence of early processing, and therefore expected to see visual dominance across all SOAs.

## 2. Experiment 1

### 2.1. Materials and Methods

#### 2.1.1. Participants

All participants gave informed consent and received course credit for their participation. Experimental procedures were approved by the University of Hawai’i Office of Research Compliance Human Studies Program (Protocol Number 2016-30820).

Twenty-seven University of Hawai’i at Mānoa undergraduate students participated in Experiment 1. One participant was removed from the sample due to failure to follow instructions, resulting in a final sample of 26 participants (18 F, 8 M; age: *M* = 20.3, *SD* = 3.31). Participants could achieve an accuracy of 74% by never making any responses to oddballs during the experiment, due to the fact that 74% of trials are standard trials in which participants make no responses. Therefore, an 80% overall accuracy (i.e., combined accuracy from all five blocks) was adopted as a benchmark for whether participants followed instructions.

#### 2.1.2. Stimuli and Apparatus

**Figure 1:**
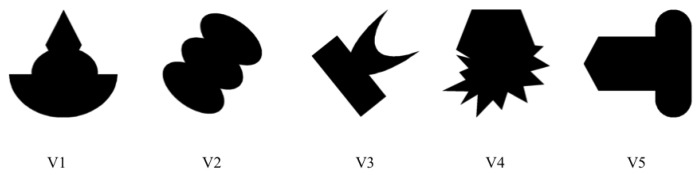
Pictures used in both experiments. In each block, one picture was selected as the visual standard and the remaining pictures were utilized as visual oddballs (see also [21]).

The visual stimuli (i.e., *pictures*) were monochromatic 400 × 400 pixel bitmap images (V1–V5; see Figure 1). The auditory stimuli (i.e., *tones*) were five pure tones that ranged from 200 Hz to 1000 Hz (A1–A5). All stimuli were previously used by Robinson et al. [21]. Pictures were presented on an Apple iMac OSX desktop computer with a monitor refresh rate of 60 Hz. Tones were heard through Logitech USB H390 headsets at a comfortable volume set by the participant. We elected to use headsets rather than speakers to minimize the distance between the participant and the audio source.

Picture-tone pairings were pseudo-randomly selected and five program versions were generated to ensure that, for each participant, each stimulus was used as the standard once (see Table 1 for a complete list of stimulus pairings).

#### 2.1.3. Procedure

**Figure 2:**
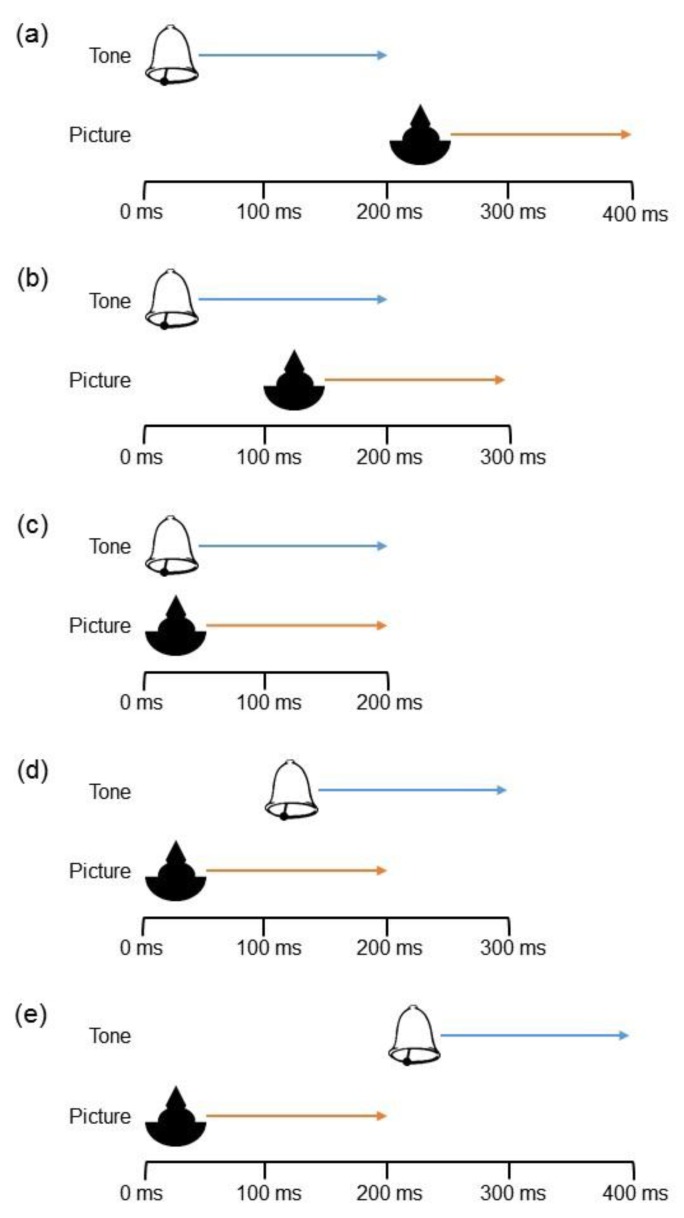
Stimulus timing. The duration of each stimulus was 200 ms. In the (**a**) +200 Auditory condition and (**b**) +100 Auditory condition, the auditory stimulus preceded the visual stimulus. In the (**c**) Simultaneous condition, the stimuli were presented at the same time. In the (**d**) +100 Visual condition and (**e**) +200 Visual condition, the visual stimulus preceded the auditory stimulus.

Every trial consisted of one visual stimulus and one auditory stimulus, each presented for 200 ms. The onset of one stimulus marked the beginning of the trial, and the onset of the other stimulus was delayed by 0 ms (control condition), 100 ms, or 200 ms. Thus, the total duration of the different trial types varied (see Figure 2). To prevent participants from being influenced by relative changes in trial length, the five stimulus timings were presented in a blocked format. Participants were randomly assigned to one of the five program versions and the order of the blocks was randomized. An intertrial interval (ITI) of 1000 ms with 15% jitter range was used (i.e., ITIs ranged from 850 ms to 1150 ms; see [28] for a discussion of optimal jitter durations).

At the beginning of each block, participants were presented with the standard picture and the standard tone, and were informed that these stimuli would co-occur frequently throughout the block. Each block lasted approximately 5 min and consisted of 188 trials. The trial ratios were generated as they were by Robinson et al. [21], with approximately 75% standard trials (140 trials with both standard stimuli), 21% unimodal oddball trials (40 trials with one standard stimulus; 20 trials with the visual standard and 20 trials with the auditory standard), and 4% bimodal oddball trials (8 trials with no standard stimuli). After each block, participants were prompted with a screen to allow a brief rest.

For our 1-button task, we chose to replicate Robinson et al.’s [21] Experiment 3 because requiring participants to not respond to bimodal oddballs ensures that they analyze both stimulus modalities conjunctively. In this task, participants were instructed to press the spacebar on the computer keyboard every time they detected a change in either the visual or the auditory stream. In the event that they detected a change in both streams, they were asked to not respond. 

Data from both experiments were deposited at the Open Science Framework and can be accessed here: https://osf.io/jhzqr/.

### 2.2. Results

For both types of unimodal oddball trials, we calculated mean response times (RTs) for correct responses and detection accuracy. Because participants could not respond until both stimuli were present, RTs were computed from the onset of the second stimulus. Detection accuracy was calculated as the number of hits divided by the number of trials of that type. For this 1-button task, RTs and accuracy cannot be calculated for bimodal oddball trials because they were identified by a non-response. Mean response times, accuracies, and test statistics for all five conditions are summarized in Table 2. 

**Figure 3:**
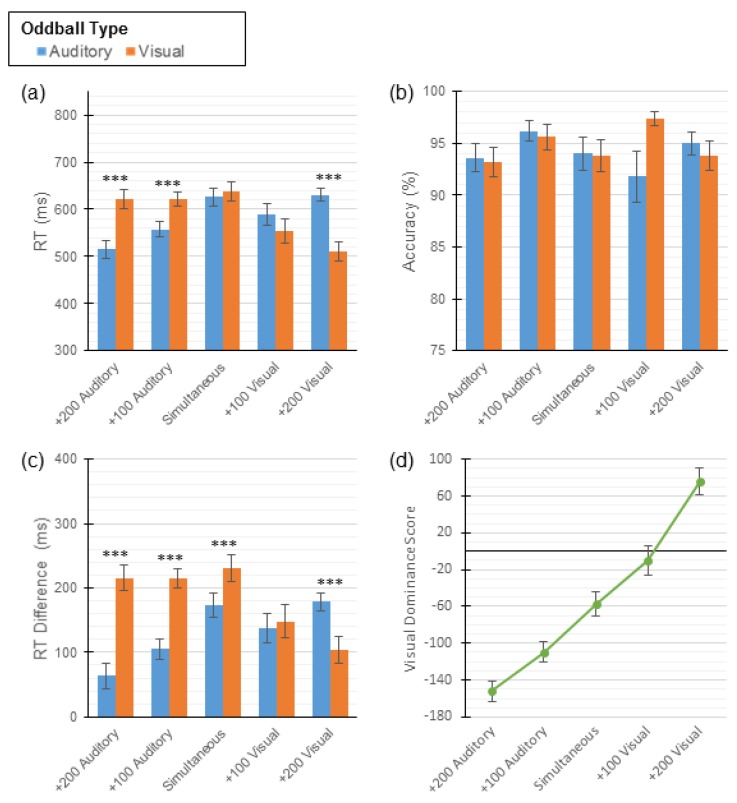
Mean scores for participants in Experiment 1. One-button task scores presented across all five conditions, by oddball type (auditory or visual, (**a**–**c**) only). Error bars show ± 1 standard error of the mean (SEM). (**a**) Mean RTs. (**b**) Mean accuracies. (**c**) Mean slowdown scores. (**d**) Mean visual dominance scores; negative values indicate auditory dominance and positive values indicate visual dominance. *** indicates *p* < 0.0002.

To assess the effect that stimulus onset asynchrony (SOA; +200 Auditory, +100 Auditory, Simultaneous, +100 Visual, +200 Visual) and oddball type (auditory, visual) had on RTs and accuracy, separate two-way repeated measures ANOVAs were conducted. For the response time data, this analysis revealed a significant main effect of SOA (*F* (4, 96) = 4.42, *p* < 0.01), no main effect of oddball type (*F* (1, 96) = 0.53, *p* = 0.48), and a significant interaction between SOA and oddball type (*F* (4, 96) = 54.17, *p* < 0.001). Paired sample t-tests comparing response times (RT) for detecting auditory oddballs and visual oddballs revealed no statistical difference in RT in the Control condition (i.e., simultaneous presentation). Across all other conditions, participants were quicker to respond to oddballs presented in the modality consistent with the stimulus that was presented first (see Table 2 and Figure 3a). A Bonferroni correction was applied to the α-levels of these and all other t-tests to control for familywise error. Each analysis involved five pairwise comparisons, so the corrected α-levels were always 0.01 (*), 0.002 (**), and 0.0002 (***).

With respect to accuracy across the five conditions, a two-way repeated measures ANOVA revealed no main effects of SOA (*F* (4, 96) = 0.85, *p* = 0.50) or oddball type (*F* (1, 96) = 0.54, *p* = 0.47); however, there was a significant crossover interaction (*F* (4, 96) = 2.92, *p* = 0.03). In the +100 Visual condition, participants made more errors identifying the auditory oddballs. In all other conditions, participants made more errors identifying visual oddballs; however, none of these differences reached statistical significance (see Table 2 and Figure 3b).

To examine the effect of interference on participants’ response times, a measure of dominance that can be easily compared across conditions was needed. *Slowdown scores* were computed by subtracting the mean RTs for unimodal oddballs from the participant’s mean RTs to oddballs. Participants in these experiments received bimodal trials only. To create slowdown scores, unimodal control data were collected from a separate sample (*n* = 42; see Appendix A for more information) and their mean scores were subtracted from the participants’ mean RTs. These means were 452 ms for auditory oddballs and 407 ms for visual oddballs. A two-way (SOA x oddball type) repeated measures ANOVA was then conducted on these scores, which demonstrated a main effect of SOA (*F* (4, 96) = 4.42, *p* < 0.01) as well as a main effect of oddball type (*F* (1, 96) = 41.66, *p* < 0.001). Additionally, a significant interaction was observed (*F* (4, 96) = 54.17, *p* < 0.001). Paired sample t-tests comparing slowdown scores by oddball type revealed auditory dominance in the Control condition (i.e., simultaneous presentation) and both Auditory conditions. In the +100 Visual condition, no difference in slowdown scores was observed. Finally, visual dominance was observed in the +200 Visual condition (see Table 3 and Figure 3c).

*Visual dominance scores* were then calculated by subtracting the visual slowdown scores from the auditory slowdown scores. In this case, values that are more negative indicate stronger auditory dominance and values that are more positive indicate stronger visual dominance. Trend analysis revealed a significant linear relationship between SOA and visual dominance score (*F* (1, 96) = 24.42, *p* < 0.001); that is, as the delay of the auditory stimulus increased, participants showed increasing levels of visual dominance (see Figure 3d).

Experiment 1 demonstrated auditory dominance shifting in accordance with the onset of the auditory stimulus, where earlier presentations of the auditory portion of the bimodal stimulus stream led to greater amounts of auditory dominance, with sensory dominance effects disappearing at a +100 visual SOA, and finally visual dominance only appearing to emerge when the visual portion of the bimodal stimulus stream preceded the auditory stimulus by 200 ms. Experiment 2 aimed to further examine the assertion that decision making is a primary component of behaviorally demonstrated sensory dominance by utilizing the same paradigm with an added decision-making component (similar to that of Robinson et al. [21], Experiment 2).

## 3. Experiment 2

### 3.1. Materials and Methods

#### 3.1.1. Participants

The recruitment procedure and inclusion criteria were the same as in Experiment 1. Twenty-nine University of Hawai’i at Mānoa undergraduate students participated in Experiment 2. Five participants were removed from the sample due to failure to follow instructions, resulting in a final sample of 24 participants (15 F, 9 M; age: *M* = 20.7, *SD* = 3.34)

#### 3.1.2. Stimuli and Apparatus

All stimuli from Experiment 1 were utilized in Experiment 2. The stimuli streams were created in the same manner described above and with the same stimulus offset timings as depicted in Figure 2.

#### 3.1.3. Procedure

The only aspect of the procedure that differed from Experiment 1 was the responses available to the participant. In this 3-button task, participants were instructed to press either the ‘1′, ‘2′, or ‘3′ key on the keyboard number pad in response to changes in the stimulus stream. For example, the ‘1′ key may have been pressed for a unimodal auditory oddball, the ‘2′ key may have been pressed for a unimodal visual oddball, and the ‘3′ key may have been pressed for a bimodal oddball. Key assignments were counterbalanced across participants.

### 3.2. Results

**Figure 4:**
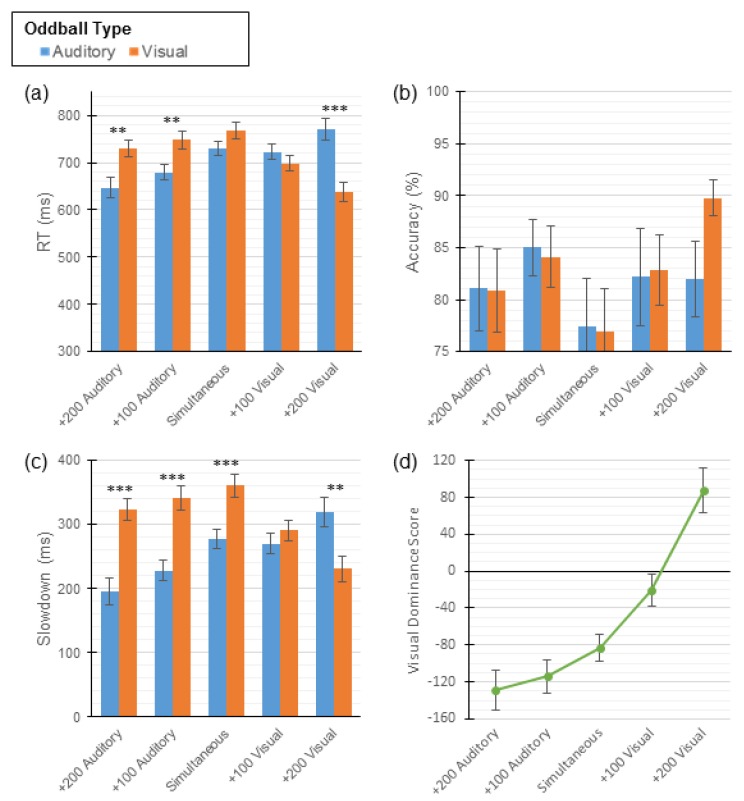
Mean scores for participants in Experiment 2. Three-button task scores presented across all Figure 1. standard error of the mean (SEM). (**a**) Mean RTs. (**b**) Mean accuracies. (**c**) Mean slowdown scores. (**d**) Mean visual dominance scores; negative values indicate auditory dominance and positive values indicate visual dominance. ** indicates *p* < 0.002, and *** indicates *p* < 0.0002.

Mean response times and accuracies were calculated as described in Experiment 1 (see Table 4). Similar to the 1-button task, of particular interest is the effect that SOA and oddball type have on both response times (RTs) and accuracy. A two-way repeated measures ANOVA with the within-participants factors of SOA and oddball type revealed a significant main effect of SOA (*F* (4, 88) = 3.78, *p* < 0.01), no main effect of oddball type (*F* (1, 88) = 0.26, *p* = 0.62), and a significant interaction between SOA and oddball type (*F* (4, 88) = 29.72, *p* < 0.001) for RTs. Paired sample t-tests comparing RTs revealed that participants were faster to detect auditory oddballs in the Control condition (i.e., simultaneous presentation). As in Experiment 1, participants were quicker to respond to oddballs presented in the modality consistent with the stimulus that was presented first; however, these differences did not reach significance in the +100 Visual condition (see Table 4 and Figure 4a).

With respect to accuracy across the five conditions, a two-way repeated measures ANOVA revealed no main effects of SOA (*F* (4, 88) = 1.43, *p* = 0.23) or oddball type (*F* (1, 88) = 0.59, *p* = 0.45), and no interaction (*F* (4, 88) = 1.72, *p* = 0.15; see Figure 4b). 

As in Experiment 1, slowdown scores were calculated (see Table 5) and used as the dependent variable in a two-way repeated measures ANOVA with SOA and oddball type as factors. The main effect of SOA was significant (*F* (4, 88) = 3.78, *p* < 0.01), as was the main effect of oddball type (*F* (1, 88) = 15.51, *p* = 0.01). Additionally, a statistically significant interaction was observed between SOA and oddball type (*F* (4, 88) = 29.72, *p* < 0.001). Paired sample t-tests comparing slowdown scores by oddball type revealed the same pattern of results as in Experiment 1: auditory dominance was observed in the control condition (i.e., simultaneous presentation) and both Auditory conditions, there was no difference in the +100 Visual condition, and visual dominance was observed in the +200 Visual condition (see Figure 4c).

Finally, in order to clarify how SOA modulated dominance across the five conditions, visual dominance scores were calculated as in Experiment 1 (see Table 5). These scores were used in a trend analysis which revealed a statistically significant linear relationship between visual dominance scores and SOA (*F* (1, 88) = 50.39, *p* < 0.001). Again, as the delay of the auditory stimulus increased, the amount of visual dominance increased (see Figure 4d). 

**Figure 5:**
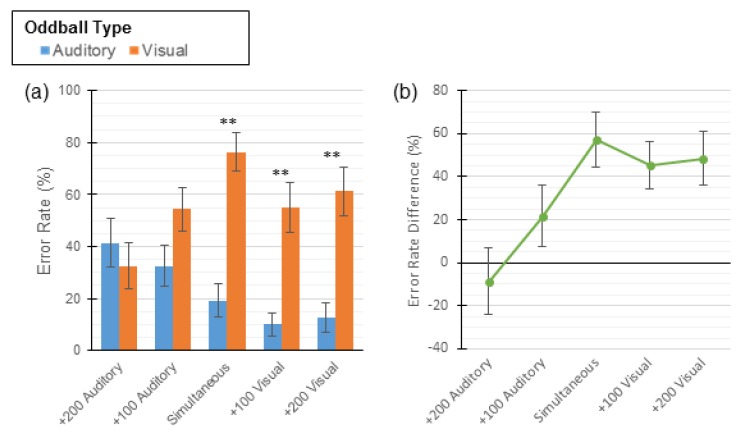
Error analysis for participants in Experiment 2. Three-button task error rates presented across all five conditions. Error bars show ± 1 standard error of the mean (SEM). (**a**) Mean error rates, by oddball type (auditory or visual). (**b**) Mean error difference scores; negative values indicate more auditory errors and positive values indicate more visual errors. ** indicates *p* < 0.002.

Unlike the 1-button task employed in Experiment 1, the 3-button paradigm allows for an in-depth analysis of participants’ errors. Error proportions during bimodal oddball trials were calculated by dividing the number of errors of each type by the participant’s total number of errors. These values were used to conduct a two-way repeated measures ANOVA with error type (auditory or visual) and SOA as IVs and the proportion of errors as the DV. The main effect of oddball error type (*F* (1, 88) = 26.78, *p* < 0.001) was significant while the main effect of SOA approached significance (*F* (4, 88) = 2.32, *p* = 0.06). Additionally, a statistically significant interaction was observed (*F* (4, 88) = 4.08, *p* = 0.004). Paired sample t-tests were conducted to identify any differences in the proportion of errors by type. Overall, participants made auditory errors and visual errors at the same rate in the +200 and +100 Auditory conditions. In the remaining conditions, visual errors were more common than auditory errors (see Table 6 and Figure 5a).

In order to fully address the significant interaction and better understand the effect that SOA had on these errors, *error difference scores* were calculated by subtracting the proportion of auditory errors made from the number of visual errors made. In this case, negative scores are indicative of more auditory errors (i.e., auditory dominance), whereas positive scores are indicative of more visual errors (i.e., visual dominance; see Table 6 and Figure 5b). Trend analysis was then conducted on these scores, revealing a statistically significant linear relationship between SOA and error difference scores (*F* (1, 88) = 12.13, *p* = 0.002). The observed relationship indicates that, as the offset of the auditory stimulus decreased, participants were more likely to press the visual oddball button during a bimodal oddball trial. In summary, the error data suggest that visual dominance is observed when the visual stimulus is presented first or simultaneously with the auditory stimulus. In contrast, when the auditory stimulus is presented first, visual dominance is eliminated, and neither modality dominates. In fact, even when the auditory stimulus led the visual stimulus by 100 ms, there was still a noticeable trend in the data in the direction of visual dominance (i.e., 21.74% increase in visual errors), although this did not reach conventional levels of statistical significance.

The final comparison that was made examined the effects of the 1-button and 3-button manipulations on visual dominance scores. It has been shown that decision making appears to have an effect on dominance, with participants showing greater amounts of auditory dominance in the 1-button task in comparison to the 3-button task [21]. Therefore, a one-way repeated measures ANOVA on visual dominance scores with task (1 or 3 buttons) as a between-subjects factor was conducted. The main effect of task was not significant (*F* (1, 46) = 0.005, *p* = 0.95); however, the main effect of SOA was significant (*F* (4, 184) = 77.56, *p* < 0.001), which was expected, given that the 1- and 3-button tasks yielded a similar pattern of results across SOA conditions. Finally, the interaction between condition and SOA was not significant (*F* (4, 184) = 0.94, *p* = 0.44).

## 4. Discussion

Colavita’s [3] finding that participants faced with a bimodal audiovisual stimulus are more likely to respond to only the visual component, while being seemingly unaware of the presence of the auditory component, has inspired much investigation. The typical finding of this research has been that visual dominance is robustly demonstrated, with very few examples of elimination or reversal of visual dominance [7,8,10]. Given the evidence accrued thus far, discovering methodologies to examine modulations of sensory dominance has become increasingly important for contemporary theorizing on sensory dominance and the developmental shift from auditory to visual dominance (e.g., [16]). With respect to this, the oddball detection paradigm utilized by Robinson et al. [21] offers an exciting opportunity to test stimulus factors that may be driving sensory dominance effects, as well as theories regarding the mechanisms which give rise to the Colavita visual dominance effect.

Our research aimed to investigate the claims that auditory dominance is more pronounced when the auditory stimulus disrupts early visual processing [1] and that, due to limited processing resources, sensory dominance may shift toward whichever modality engages those processing resources first [13]. We conducted two experiments using the oddball detection task; Experiment 1 employed the 1-button version, and Experiment 2 used the 3-button version. In both experiments, stimulus onsets were manipulated such that the presentation of the second stimulus was delayed by 0, 100, or 200 ms. Based on the results of Robinson et al. [21] we expected that, in the 1-button task (Experiment 1), auditory dominance would be observed in the simultaneous (control), +100 Auditory, and +200 Auditory conditions, and that visual dominance would be observed in the +100 Visual and +200 Visual conditions. Additionally, in the 3-button task (Experiment 2), we predicted that visual dominance would occur in all conditions due to the increase in the number of possible responses.

When comparing the slowdown scores, the expected effect of presentation order in the 1-button task was supported overall. In both experiments, auditory dominance was observed in the simultaneous presentation, +100 Auditory, and +200 Auditory conditions; no dominance was observed in the +100 Visual condition; and visual dominance was observed in the +200 Visual condition. The results from these last two conditions are especially important because they demonstrate the strength of auditory dominance. In this case, elimination of auditory dominance only occurred when the visual stimulus was presented 100 ms prior to the auditory stimulus (+100 Visual condition), and reversal to visual dominance was only observed when the visual stimulus was presented well in advance (+ 200 Visual condition). Trend analysis further revealed a positive linear relationship between visual dominance scores and the delay of the auditory stimulus.

Regarding Experiment 2, both the auditory dominance observed in the simultaneous condition and the lack of dominance in the +100 Visual condition were surprising, given the results of previous experiments using this 3-button task (see [21]). Because the same pattern of dominance was observed across both tasks, as assessed by slowdown scores, the number of response options does not appear to affect patterns of sensory dominance when manipulating stimulus timing, which may suggest that these interference effects are happening before the decision/response phase (but see [29], which did not find cross-modal interference when looking at cardiac responses to oddballs). 

Additionally, Experiment 2 allows for an assessment of the errors made by participants during bimodal oddball trials. When the auditory stimulus preceded the visual stimulus, there was no difference in the proportion of auditory- or visual-only errors, indicating an elimination of visual dominance. In the remaining conditions, visual dominance was observed. These findings are consistent with the analysis of the slowdown scores, except for the simultaneous presentation condition. In other words, the type of dominance demonstrated in this condition depends on how dominance is being measured. Although a puzzling combination of results, it does provide support for Robinson and Sloutsky’s [22] argument that auditory dominance is more likely to be observed in more implicit measures of dominance (e.g., the effect of interference between modalities on visual dominance scores) than in more explicit measures of dominance (e.g., errors in button presses).

The results of Experiment 2 support the hypothesis that early entry into processing might modulate sensory dominance; namely, that whichever modality enters processing first is the de facto winner of the race for processing resources and, as a result, modulates sensory dominance. More specifically, in both the 1-button and 3-button tasks, auditory dominance was robustly demonstrated in the RT data in the +200 Auditory, +100 Auditory, and simultaneous presentation conditions, with no dominance type being demonstrated in the +100 Visual condition, and visual dominance being demonstrated in the +200 Visual condition. A potential neural explanation for these results lies in the difference in transmission speeds within auditory and visual sensory pathways. In general, it has been shown that auditory input arrives at the sensory cortex more rapidly than does visual input. However, these differences in cortical response latency are largely eliminated by the time the signals reach higher cortical areas involved in multisensory processing [30]. As a result, although important to note, we do not believe this difference in transmission times accounts for our findings here.

As discussed earlier, the amount of lead time needed for the visual stimulus to elicit visual dominance matters in the 3-button task. However, this was not the case in the 1-button condition, wherein a positive relationship between the lead on the auditory stimulus and the amount of auditory dominance was observed. With respect to the +100 Visual condition, no dominance was demonstrated in either task. It is possible to conclude that, in this paradigm, the prepotency of auditory dominance requires that the visual stimulus have a 100-ms lead before processing resources are equally distributed to both modalities (see [31] for a similar pattern of results). This interpretation supports the claim that auditory stimuli are prioritized in early processing, to such a degree that this lead is necessary to negate these inherent processing biases. However, when given the lead time of 200 ms, visual dominance becomes more apparent in both versions of the task. 

Although this pattern of results seems to indicate shifting dominances during both the 1-button and 3-button SOA manipulations, it is again important to highlight the results of the error data in the 3-button condition (Experiment 2), which demonstrates clear evidence of visual dominance in the simultaneous presentation and +100 and +200 Visual conditions. As discussed previously with respect to Experiment 1, this pattern of results is unique and interesting in that it demonstrates that the type of dominance observed can depend on how dominance is measured. In effect, the pattern of results can be interpreted in two meaningful ways: (1) that early processing favors the auditory modality and later processing favors the visual modality, or (2) that, when the response requires a decision, there is a behavioral pre-potency toward responding to visual stimuli over auditory stimuli. Indeed, using a similar oddball detection paradigm, Chandra, Robinson, and Sinnett [26] showed that participants were more likely to make visual errors during bimodal trials, lending credence to the latter interpretation of these results.

Finally, it has been argued that processing resources are actively shared across both the auditory and visual modality and, as a result, this finite resource is taxed such that allowing a modality to engage those resources first results in dominance of that modality. One could argue that multisensory processing resources are not engaged until both modalities have received sensory information to process. One key assumption of this argument is that, in order for multisensory processing resources to be engaged, the unisensory components of the bimodal signal must be presented simultaneously [32,33]. By this account, it would be difficult for manipulations to SOA to provide insight regarding the role of stimulus features in multisensory processing. However, estimates of the maximum offset at which multisensory integration still occurs range from 300 ms [34,35] to 1000 ms [36] –100 to 800 ms longer than any of the SOAs we used.

However, Koppen and Spence [14] demonstrated that the temporal window of audiovisual integration can occur at a 65 ms lead for the auditory modality and 89 ms lead for the visual modality. While our manipulations fall outside of this window, with the obvious exception of the simultaneous condition, it could still be argued that the +100 Visual and +100 Auditory conditions may have been subject to integration. If this were to be the case, then this could account for the lack of dominance demonstrated in the +100 Visual condition. Interestingly, Koppen and Spence [14] demonstrated that the Colavita visual dominance effect occurs within the aforementioned temporal window of multisensory integration. The conclusion that the Colavita effect occurs after audiovisual events are combined into a unitary percept is further supported by the error data collected in Experiment 2, wherein we observed more visual errors than auditory errors in all conditions other than the +200 Auditory condition (see Figure 5). Nevertheless, we did not calculate a possible window of integration for our participants; therefore, future research will need to systematically examine the relationship between individual differences in the window of integration and patterns of modality dominance.

When examining the results of the simultaneous presentation condition of the 3-button task (Experiment 2), the slower responses to visual oddballs is important to note because it opposes the findings of Robinson et al.’s [21] Experiment 3. It is difficult to determine whether these divergent findings are a result of sampling error, or instead due to methodological differences described earlier. As a result, two methodological issues occur that warrant potential future investigation. First, participants in Robinson et al.’s [21] 3-button condition made one response on unimodal control trials, and multiple responses to the bimodal stimulus stream. In contrast, the 1-button condition compares single response unimodal controls to single responses during the bimodal stimulus streams. It is possible that this difference may influence observed dominance types when they are measured by relative slowdowns between unimodal and bimodal response times. Therefore, better controls for the 3-button oddball detection task should be considered. Secondly, as was discussed briefly, it is important to understand whether participants view the bimodal stimulus streams as a single unitary percept, and as of now the paradigm does not have a way to meaningfully determine if this is occurring. This issue may be exacerbated in the 3-button condition, in that participants are monitoring each stimulus stream and making a different response for the oddballs in the auditory and visual streams. Therefore, it is possible that multisensory integration may not be occurring as deeply as it does in the 1-button condition or, alternatively, that sensory dominance effects can occur without multisensory integration.

One possible weakness of this research is that, unlike Robinson et al. [21], unimodal controls were obtained between-subjects rather than within-subjects. This design choice was made due to concerns about participant fatigue. However, such unimodal controls would allow for additional ways to look at sensory dominance (i.e., via relative slowdown rates for the bimodally presented auditory and visual stimuli); therefore, a fully within-subjects design is an important future step in this research.

Overall, the aim of this research was to assess the effect that early entry into processing has on sensory dominance. It was found that manipulations to SOA, and thus early entry into processing, did exert a strong effect on dominance. In general, participants showed shifts in dominance in accordance with which stimulus occurred first; however, the visual stimulus needed a larger lead time for the effect to manifest. These findings support the claims made by others that early entry into processing affects sensory dominance, and that the auditory modality may be especially favored during these early stages due to the typically transient nature of naturally occurring auditory stimuli [13].

## Figures and Tables

**Table 1:** Standard stimuli by program version and block. Within each program version, each auditory stimulus (A1–A5) and each visual stimulus (V1–V5) was used as a standard in exactly one block.

	+200 Auditory	+100 Auditory	Simultaneous	+100 Visual	+200 Visual
Version 1	A1, V1	A3, V4	A2, V5	A4, V2	A5, V3
Version 2	A2, V5	A1, V1	A5, V3	A3, V4	A4, V2
Version 3	A5, V3	A2, V5	A4, V2	A1, V1	A3, V4
Version 4	A4, V2	A5, V3	A3, V4	A2, V5	A1, V1
Version 5	A3, V4	A4, V2	A1, V1	A5, V3	A2, V5

**Table 2:** Reaction times and accuracy scores in the 1-button task. Means, standard deviations, and *t* statistics from pairwise comparisons of auditory and visual oddballs.

	RT (ms)			Accuracy (%)		
SOA Condition	Auditory M (SD)	Visual M (SD)	*t* Score	Auditory M (SD)	Visual M (SD)	*t* Score
+200 Auditory	515 (97.26)	622 (99.54)	−9.31 ***	93.6 (6.70)	93.2 (6.90)	0.32
+100 Auditory	557 (79.95)	622 (77.06)	−5.82 ***	96.2 (4.85)	95.6 (6.18)	0.53
Simultaneous	626 (95.05)	638 (103.96)	−0.96	94.0 (8.04)	93.8 (7.54)	0.10
+100 Visual	590 (117.78)	555 (130.69)	2.15	91.8 (12.15)	97.4 (3.26)	2.54
+200 Visual	631 (67.53)	510 (103.33)	8.32 ***	95.0 (5.40)	93.8 (7.11)	0.77

Note: Significance was assessed with *df* = 24. *** indicates *p* < 0.0002.

**Table 3:** Auditory dominance in the 1-button task. Means and standard deviations, and *t* statistics from pairwise comparisons of slowdown scores for auditory and visual oddballs. Means and standard deviations of visual dominance scores; negative values indicate auditory dominance and positive values indicate visual dominance.

	Slowdown (ms)			Dominance
SOA Condition	Auditory M (SD)	Visual M (SD)	*t* Score	M (SD)
+200 Auditory	63 (97.26)	215 (99.54)	−13.22 ***	−152 (57.51)
+100 Auditory	105 (79.95)	215 (77.06)	−9.88 ***	−110 (55.40)
Simultaneous	174 (95.05)	231 (103.69)	−4.52 ***	−57 (63.29)
+100 Visual	138 (117.78)	148 (130.69)	−0.63	−10 (80.93)
+200 Visual	179 (67.53)	103 (103.33)	5.21 ***	75 (72.42)

Note: Significance was assessed with *df* = 24. *** indicates *p* < 0.0002.

**Table 4:** Reaction times and accuracy scores in the 3-button task. Means, standard deviations, and *t* statistics from pairwise comparisons of auditory and visual oddballs.

	RT (ms)			Accuracy (%)	
SOA Condition	Auditory M (SD)	Visual M (SD)	*t* Score	Auditory M (SD)	Visual M (SD)
+200 Auditory	647 (104.11)	730 (88.74)	−3.87 **	81.1 (19.42)	80.9 (19.40)
+100 Auditory	680 (80.47)	749 (94.14)	−3.78 **	85.0 (13.14)	84.1 (14.27)
Simultaneous	730 (76.95)	768 (90.36)	−2.61	77.4 (22.20)	77.0 (19.93)
+100 Visual	722 (82.20)	698 (82.07)	1.43	82.2 (22.60)	82.8 (16.36)
+200 Visual	771 (114.65)	638 (101.05)	5.34 ***	82.0 (17.56)	89.8 (8.32)

Note: Significance was assessed with *df* = 22. ** indicates *p* < 0.002, and *** indicates *p* < 0.0002.

**Table 5:** Visual dominance scores in the 3-button task. Means and standard deviations, and *t* statistics from pairwise comparisons of slowdown scores for auditory and visual oddballs. Means and standard deviations of visual dominance scores; negative values indicate auditory dominance and positive values indicate visual dominance.

	Slowdown (ms)			Difference (ms)
SOA Condition	Auditory M (SD)	Visual M (SD)	*t* Score	M (SD)
+200 Auditory	195 (104.11)	323 (88.74)	−5.96 ***	−128 (103.33)
+100 Auditory	228 (80.47)	342 (94.14)	−6.25 ***	−114 (87.37)
Simultaneous	278 (76.95)	361 (90.36)	−5.71 ***	−83 (69.64)
+100 Visual	270 (82.20)	291 (82.07)	−1.23	−21 (80.94)
+200 Visual	319 (114.65)	231 (101.05)	3.28 **	88 (119.01)

Note: Significance was assessed with *df* = 22. ** indicates *p* < 0.002, and *** indicates *p* < 0.0002.

**Table 6:** Error rates and error rate difference scores in the 3-button task. Means and standard deviations of rate of incorrect responding to bimodal oddballs, by type of error, and *t* statistics from pairwise comparisons. Means and standard deviations of error rate differences.

	Error Rate (%)			Difference (%)
SOA Condition	Auditory M (SD)	Visual M (SD)	*t* Score	M (SD)
+200 Auditory	41.30 (44.63)	32.61(41.89)	0.56	8.70 (74.00)
+100 Auditory	32.61 (36.83)	54.35 (40.66)	−1.50	−21.74 (69.53)
Simultaneous	19.28(30.71)	76.38 (34.68)	−4.41 **	−57.10 (62.10)
+100 Visual	10.04 (21.73)	55.18 (45.97)	−4.09 **	−45.13 (52.91)
+200 Visual	12.68 (27.28)	61.23 (45.48)	−3.88 **	−48.55 (60.07)

Note: Significance was assessed with *df* = 22. ** indicates *p* < 0.002.

## Appendix A. Unimodal Control Methodology

Recall that Robinson et al. [21] theorized that modality dominance is demonstrated in these oddball tasks by whichever modality is affected less by the presence of the other in the bimodal stimulus stream, and this would ultimately be reflected in the differences in response times for auditory and visual oddballs in the unimodal and bimodal stimulus streams. As a result, a sample of participants was assigned to unimodal auditory or visual control conditions and their response times were utilized as a baseline for calculating dominance scores in each experiment. Herein is a description of the methodology utilized to conduct these unimodal controls.

### Appendix A.1. Participants

Forty-two University of Hawai’i at Mānoa undergraduate students participated in this experiment (29 F, 13 M; age: *M* = 21.00, *SD* = 5.47).

### Appendix A.2. Stimuli and Procedure

Stimuli consisted of the same five visual stimuli and five auditory stimuli used in Experiments 1 and 2. Participants were assigned to one of two possible conditions, a unimodal auditory condition or a unimodal visual condition; therefore, each participant either saw pictures or listened to tones. Participants were randomly assigned a standard (one of the five auditory stimuli if they were in an auditory condition or one of the five visual stimuli if they were in a visual condition). The stimulus stream consisted of 360 trials in total, and of which 80 (20%) were oddballs (i.e., 20 of each non-standard stimulus). Participants were instructed to press the spacebar on the computer every time they either saw an image or heard a sound that was different from their standard. Participants’ response times and responses were recorded.
